# Lead-Based Decorative Paints: Where Are They Still Sold—and Why?

**DOI:** 10.1289/ehp.122-A96

**Published:** 2014-04-01

**Authors:** Rebecca Kessler

**Affiliations:** Rebecca Kessler is a science and environmental journalist based in Providence, RI.

_In 20_02 researchers at South Africa’s Medical Research Council collected blood from first-graders in impoverished townships of Johannesburg to check their exposure to lead, a powerful neurotoxicant. The children’s blood lead levels were high by today’s standards, averaging 9 µg/dL.^1^ But one student had 52 µg/dL of lead coursing through her veins, far above the 5-µg/dL concentration at which intervention is currently recommended in the United States.^2^ The researchers went to her apartment to investigate and met a skinny, withdrawn little girl and her parents.

**Figure:**
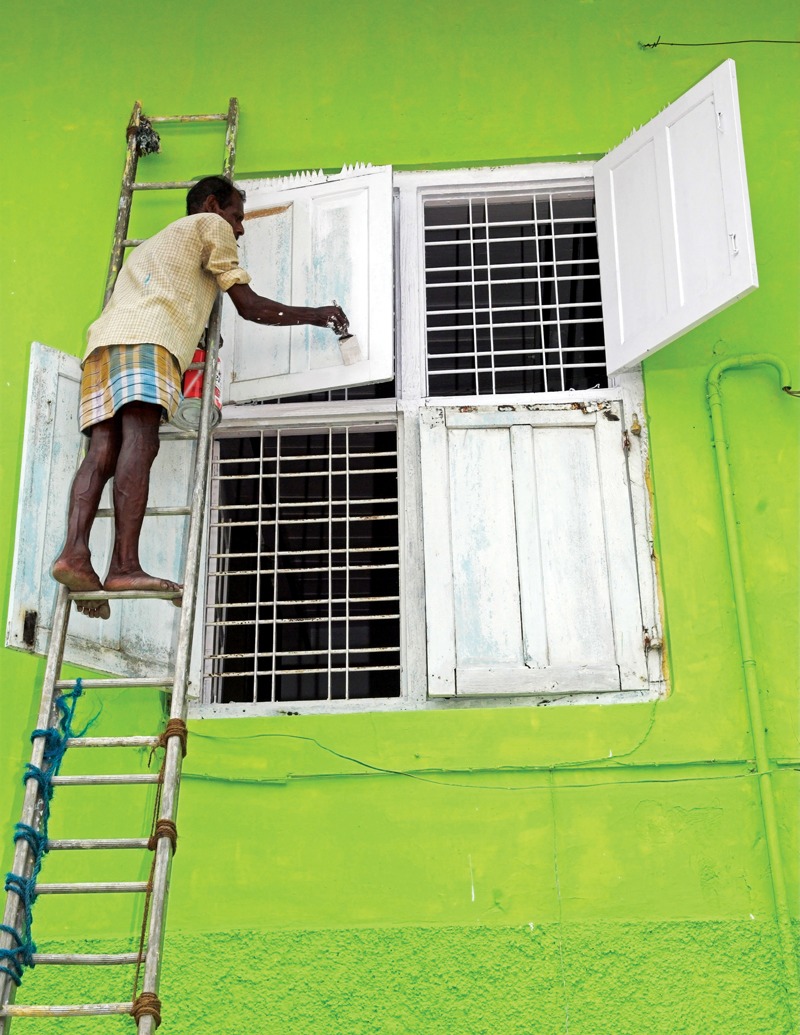
**What Are Decorative Paints?** In the context of lead-based paint regulations, the term “decorative” typically refers to paints used on indoor or outdoor walls of homes and other architectural structures. It also refers to paints sold to consumers for household use, such as anti-corrosion products used on bicycles, windows, gates, and other metal surfaces. Industrial paints are used for all other applications, including automotive and other coatings, structural paints to inhibit metal corrosion, and marine paints. Although the paints used on toys are technically industrial paints, legislation such as the Philippines’ recent Chemical Control Order separately bans lead in the manufacture of toys, school supplies, and other consumer products.^38^ © Charles Stirling/Alamy

“You’re here because of what she eats,” study leader Angela Mathee recalls the girl’s mother saying. Huge patches of pale lemon-yellow paint were missing from every wall in the apartment, where the girl had spent hours chipping it away and eating it. The windowpanes were loose because she had eaten the painted putty holding them in place, and the dirt outside was pitted where she had devoured it, too. Carmelita (a pseudonym) had severe pica, the compulsive consumption of nonfood substances. Her parents had taken her to various doctors, but they had offered little help. None had tested her blood, although lead poisoning often shadows pica like a phantom.

When Mathee met Carmelita, it had been decades since developed nations had banned lead-based paint, and South African paint companies had long before voluntarily agreed to abandon lead, too.^3^ But when Mathee tested paint from the apartment walls it was loaded with lead, even though Carmelita’s parents said they’d purchased it recently.^1^^,^^3^

While pica presents a dramatic example of exposure to lead, far more children can be dangerously exposed just by inadvertently consuming dust from deteriorating paint through normal activity.^4^^,^^5^ Often, lead exposure has no observable symptoms and goes unrecognized.^6^ But early exposure can cause profound neurobehavioral problems including decreased life-long intellectual performance and behavioral changes—even at blood concentrations below 5 µg/dL, which researchers once thought far too low to harm kids.^5^^,^^7^ Lead exposure has also been associated with Alzheimer’s disease and cardiovascular disease.^8^^,^^9^

Disturbed by what they found in Carmelita’s apartment, Mathee’s team surveyed homes across Johannesburg. They discovered that 20% of the homes sampled, both old and new, rich and poor, had lead-based paint on the walls.^3^ Most of the colored oil-based household paints for sale in stores contained lead, too, often at concentrations thousands of times above the current U.S. standard of 90 ppm.^10^ When the investigators tested paints on children’s toys, they again found lead. Mathee was horrified to discover levels as high as 135,000 ppm on toys in her own home—including building blocks bearing her young daughter’s tooth marks.

“At that time I had been working on lead issues for nearly two decades, and it struck home that unless there are protective measures in place … none of us, no matter how much you know, can protect your children against this public health hazard,” Mathee says. “We have to put in place broad measures, regulatory measures, to protect everyone.”

The team’s evidence convinced the government to ban lead in household paints, effective in 2010.^11^ Mathee’s team helped Carmelita get treatment and had her apartment remediated, but the girl continued to struggle with pica, and eventually they lost track of her. She would be about 19 now, Mathee guesses. “Had it not been for Carmelita, we probably wouldn’t have paint lead regulations in place in the country now,” Mathee says. “The South African public owes her a debt of gratitude.”

Even so, South Africa still has a long way to go. Subsequent testing by Mathee’s team shows that lead-based paints are still widely sold, despite calls by researchers and South Africa’s main paint manufacturers association for the government to start prosecuting companies that violate the law.^12^ In addition, Mathee says, many doctors remain unaware of the extent of lead exposure in children, and the country lacks fundamental infrastructure and systems to diagnose and treat lead poisoning. There are no childhood blood lead standards or any national surveillance programs in place in South Africa to reveal how many children are exposed countrywide. Public awareness of lead hazards is low, she says, and most people don’t know that lead-based paint could be in their homes, let alone how to safely maintain or renovate painted surfaces.

For all that, South Africa is a step ahead of most developing nations. Rising incomes have enabled more and more people to afford a splash of color in their lives, with booming sales in decorative paints used on homes, furniture, toys, and more.^13^ Yet few of these countries regulate lead in paint at all. And paints loaded with lead are readily available on store shelves, rarely bearing any labeling to warn consumers of the dangers they pose, a spate of recent studies shows.^14^ Now, however, an international effort is gathering steam to remove lead from paints once and for all.

## Getting the Lead Out

Lead compounds are typically added to oil-based enamel paints as pigments, or to improve opacity and durability.^14^^,^^15^^,^^16^ Lead-based paints have been implicated in children’s poisonings since at least 1904, when lead toxicity in several Australian children was traced to disintegrating lead-based paint on the porches of their homes.^17^ Within a few years, several nations in Europe and elsewhere began banning lead in certain household paints.^4^^,^^5^^,^^18^

In the United States, a voluntary standard limited lead in interior paints beginning in 1955. But the country did not ban lead-based consumer paints outright until 1977, when it capped the allowable concentration at 600 ppm, or 0.06% of the weight of the total nonvolatile content of the paint. In 2009 that limit dropped to 90 ppm.^19^ Nevertheless, the issue remains alive in U.S. homes and courtrooms, with a costly ongoing effort to make millions of old homes safe for children, and legal battles seeking money to pay for remediation from companies that once sold lead-based paints.

By several accounts, many U.S. public health workers who had been diligently working to reduce lead exposure simply assumed the rest of the world had acted, too. This community was taken by surprise as reports starting trickling out around 1999 that lead-based decorative paints were still being manufactured abroad.^14^ Then came 2007, when news broke that Asian toys imported to the United States and Europe were coated with high-lead paint, sparking public ire.^20^

“This posed an obvious question—what about paints being sold in Asia for Asians? There was very little attention given to that,” says Jack Weinberg, senior policy advisor at the International POPs Elimination Network (IPEN), a coalition of environmental and health groups that has been testing the lead content of decorative paints for sale in numerous developing nations and working to get lead-based paint banned. Belarus, Brazil, China, India, Malaysia, Mexico, Nigeria, Tanzania, and beyond—everywhere researchers looked, enamel paints with striking levels of lead were being sold freely.^21^^,^^22^^,^^23^^,^^24^

As for how this could be, so long after wealthier countries abandoned lead in residential paints, Weinberg says inertia is largely to blame. “Nobody was paying attention,” he says. Lead-based pigments are marginally cheaper for some products, he explains, but mainly they’ve simply been around for a long time, are easy to produce, and are widely available. “Some argue lead pigments are more durable, more protective, or have better colors, but these claims are highly debatable and, I think, don’t hold up,” Weinberg says. “In the absence of a legal requirement, a lot of companies just do it.”

In 2009, at the second International Conference on Chemicals Management in Geneva, representatives of more than 120 countries voted to support a global partnership to phase out lead-based paints and tasked the United Nations Environment Programme (UNEP) and the World Health Organization (WHO) with organizing the effort.^25^ The partnership, called the Global Alliance to Eliminate Lead Paint (GAELP), launched in 2010.^26^ Members include IPEN and other citizen groups, a paint industry group, and government agencies from the United States, Honduras, Cameroon, Paraguay, and Switzerland. Toward the goal of eliminating lead in paints by 2020, alliance members raise public awareness, encourage governments to pass regulations, and educate paint companies about suitable alternatives to lead.^27^

**Figure:**
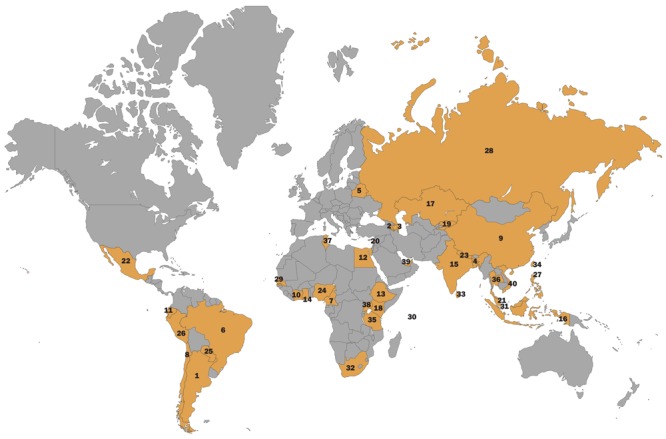
**Where Are Lead-Based Decorative Paints Still Sold?** 1 Argentina2 Armenia 3 Azerbaijan4 Bangladesh5 Belarus6 Brazil7 Cameroon8 Chile9 China 10 Côte d’Ivoire11 Ecuador12 Egypt13 Ethiopia 14 Ghana15 India16 Indonesia17 Kazakhstan18 Kenya19 Kyrgyzstan20 Lebanon21 Malaysia22 Mexico23 Nepal24 Nigeria25 Paraguay26 Peru27 Philippines28 Russia29 Senegal30 Seychelles31 Singapore32 South Africa33 Sri Lanka34 Taiwan35 Tanzania36 Thailand37 Tunisia38 Uganda^33^39 United Arab Emirates^34^40 Vietnam^35^ © Charles Stirling/Alamy

In October 2013 GAELP members released a trove of new data in conjunction with an international public awareness campaign. One installment was a UNEP-funded study and report carried out by IPEN and its local partners that detailed lead-testing results from 234 cans of enamel decorative paints purchased in nine countries: Argentina, Azerbaijan, Chile, Côte d’Ivoire, Ethiopia, Ghana, Kyrgyzstan, Tunisia, and Uruguay.^14^ Paints with greater than 10,000 ppm of lead were identified in all the countries but Chile and Uruguay, and paints with greater than 99,000 ppm turned up in Argentina, Côte d’Ivoire, Ethiopia, and Tunisia.

Chile and Uruguay were bright spots. These countries had banned paints with lead above 600 ppm,^28^^,^^29^ and indeed, most of their paints contained low levels. Argentina has a similar ban^30^ but still had high-lead paints on store shelves. Nevertheless, the report concluded that regulations can work. Separate reports from IPEN partners in Paraguay^31^ and Russia^32^ bring to at least 40 the number of countries in which lead-based decorative paints have recently been documented.^14^^,^^33^^,^^34^^,^^35^

IPEN and its partners in its Asian Lead Paint Elimination Project just released a new report on seven Asian countries where they had previously discovered lead-based paints and begun pushing to eliminate them: Bangladesh, India, Indonesia, Nepal, the Philippines, Sri Lanka, and Thailand.^36^ Although lead-based paints were still widely sold in each of these countries, several of the region’s large paint companies apparently eliminated lead across their decorative paint lines. Sri Lanka and the Philippines enacted mandatory regulations limiting lead in paints, and Bangladesh, Nepal, and Indonesia are considering how best to do so. Thailand and India set voluntary standards.^37^

## Shifting the Paint Industry

The Philippines’ regulation, enacted in December 2013, sets a 90-ppm standard for lead in decorative paints by 2016 and in industrial paint by 2019.^38^ The paint industry, government, and nongovernmental organizations (NGOs) are also setting up a third-party certification program to verify that paints meet the standard, according to Johnson Ongking, vice-president of one of the country’s largest paint companies, Boysen Paints. Ongking, a recent president of the Philippines Association of Paint Manufacturers, says the country’s 23 main paint companies will be ready to comply.

According to Ongking, Boysen eliminated lead around 2007, and its cans now carry an icon advertising their lead-free status. He says the process took two years to complete and entailed a price increase of 10–30% for affected paints (a small portion of the company’s product line).

Ongking says IPEN and its Filipino partner, EcoWaste Coalition, brought the dangers of lead to the industry’s attention. The two groups were helpful in educating companies about the need and means for reformulating their paints as well as in crafting regulation. “Honestly, we just weren’t that aware of the hazards of lead in paint,” he says. “The more we learned and understood about the health risks involved … it really was kind of a no-brainer.”

Ultimately, Ongking says, eliminating lead industry-wide will be good for the paint business and will earn customers’ trust. “It gives them confidence that we’re responsible as an industry, that we look after what’s good for our consumers,” he says.

Nepal faces different challenges. The Kathmandu-based Center for Public Health and Environmental Development, an IPEN partner and GAELP member, conducted studies of lead in paints in 2010, 2011, and 2013, each time turning up plenty of high-lead paints on store shelves.^39^ The group publicized its results at every step through media campaigns that elicited a strong public reaction, says executive director Ram Charitra Sah. It raised the issue with the government, pediatricians, and the school system, and began lobbying for regulation.

**A Selective Timeline of Lead-Based Paints**

300 BCE—Theophrastus describes the preparation of “white lead,” a tintable powder used as a base for paint, using vinegar and lead metal.^57^
1622—The Dutch process, the oldest commercial method for producing white lead, has become established in the Netherlands.^58^ This process is based on the reaction described by Theophrastus.
1804—The first U.S. white lead factory is established in Philadelphia.^59^
1848—French physician Louis Tanquerel des Planches writes a treatise on toxicity resulting from exposure to lead in paint.^60^
1891—National Lead Company is incorporated. This company will dominate the production and sale of lead products in the United States throughout the twentieth century.^61^
1904—J. Lockhart Gibson is among the first to identify lead-based paint as a source of child lead toxicity in his report in the Australasian Medical Gazette.^17^
1909—Austria, Belgium, and France become the first countries to ban lead-based interior paints.^18^
1914—Physicians H.M. Thomas and K.D. Blackfan report the first documented U.S. case of child lead poisoning attributed to paint ingestion.^62^
1922–1934—Lead-based interior paints are banned in Greece, Tunisia, Czechoslovakia, Great Britain, Sweden, Belgium, Poland, Spain, Yugoslavia, and Cuba.^18^
1943—Pediatricians Randolph Byers and Elizabeth Lord publish the first quantitative analysis of neurodevelopmental impacts of lead exposure.^63^
1955—The American National Standards Institute adopts a voluntary standard stipulating that lead constitute less than 10,000 ppm of the total weight of solids in interior paints.^64^
1971—President Richard Nixon signs the Lead-Based Paint Poisoning Prevention Act. At this time an estimated 6–28% of urban children have blood lead levels greater than 50 µg/dL.^65^
1977—The U.S. Consumer Product Safety Commission limits lead in paints for residential use to 600 ppm.^66^
1990—By now most highly industrialized countries have regulated lead-based interior paints to some degree.^14^ The U.S. Department of Housing and Urban Development issues interim guidance, its first, for abatement of lead-based paint hazards in public housing.^65^
1997—Australia reduces the maximum lead content of residential paints to 1,000 ppm,^68^ and Chile limits lead in decorative paints to 600 ppm.^28^ The American Public Health Association issues a policy statement titled “Responsibilities of the Lead Pigment Industry to Support Efforts to Address Lead Poisoning.”^69^
2002—The World Summit on Sustainable Development adopts a resolution to phase lead out of paints.^70^
2009—The International Conference on Chemicals Management identifies lead-based paints as a priority policy issue.^25^ South Africa limits lead in decorative paints to 600 ppm.^11^ The United States adopts its current limit of 90 ppm lead in paints intended for consumer use.^19^
2010—The Global Alliance to Eliminate Lead in Paint is established by the World Health Organization and the United Nations Environment Programme.^26^
2011—Uruguay limits lead in decorative paints to 600 ppm.^29^
2013—Sri Lanka limits lead in decorative paints to 600 ppm,^36^ and the Philippines limits lead content to 90 ppm.^38^ In the United States, California judge James Kleinberg finds three companies liable for creating a public nuisance by promoting the use of lead-based interior paints for decades after they were recognized as potentially harmful. In the 7 January 2014 final ruling, Sherwin-Williams, NL Industries (formerly National Lead Company), and ConAgra are ordered to pay $1.15 billion into a fund to remove lead-based paint from homes in California.^71^

“Things have changed a lot,” Sah says. Now schools—where children spend their days at brightly colored desks and benches—are shifting toward safe paints, and the government is drafting regulations limiting lead in paints to 90 ppm. Sah is optimistic that lead-based paints’ days are numbered in Nepal.

But Nepalese paint manufacturers still see a difficult road ahead. While acknowledging the health hazards posed by lead, Bishwa Prakash Saakha, president of the Nepal Paint Manufacturers Association, points to several obstacles that won’t be overcome just by penning a regulation. A lot of paints from neighboring India enter the country unofficially, so unless India enacts and enforces a mandatory lead-paint ban of its own, any domestic regulation will do Nepal little good, he says. And enforcement of any new law will be essential but difficult, with only a handful of laboratories in the country capable of testing for lead.

Most of all, Saakha says, Nepalese paint manufacturers need help reformulating their paints, as most rely on old formulas and are unaware even of which pigments contain lead and which do not. “It’s not that the paint manufacturers association doesn’t want to go for the lead-free paint. We want to. But it will take time,” Saakha says, adding that the company he represents, Nepal Paints, is trying to reformulate now. “We are working on this, but it is difficult for us,” he says.

GAELP is developing guidance for countries interested in regulating lead-based paints. Given the varying needs around the world, the guidance will likely include a menu of regulatory approaches and options for enforcement, says Angela Bandemehr, an international environmental program coordinator with the U.S. Environmental Protection Agency (EPA), which chairs the alliance’s advisory committee. Bandemehr says the goal is to enable countries to share information and learn from one other what works. “We want to empower countries to look at their own situation and do what’s best for them to do,” Bandemehr says. “It’s not a one-size-fits-all situation.”

## The Cost of Reformulating

A common refrain from manufacturers is that the cost of eliminating lead will be prohibitive, potentially putting them out of business, and that consumers will have to pay more. However, the UNEP report found that paints with and without lead are both sold almost everywhere at comparable prices. Boysen’s 10–30% price increase notwithstanding, the report notes that an informal survey of manufacturers indicates minimal increases in material costs and unaffected sales prices. Rather, it’s the time and effort to reformulate paint recipes that typically pose the greatest challenge, particularly for smaller companies.^14^

Any costs are likely to be negated by paint companies having increased access to markets where lead is restricted, says Steve Sides of the International Paint and Printing Ink Council (IPPIC), an association of international trade associations and a GAELP member. Sides notes that IPPIC members come mainly from industrialized countries that already have restrictions on lead use in paints. Calling lead-based paint “an archaic technology,” Sides says IPPIC strongly supports the alliance’s overall goals, in particular the need for regulation to create a level playing field among paint companies.

With some exceptions, Weinberg says, companies have generally continued producing lead-based paints “until faced with an active national effort that makes this an issue they can no longer avoid.” By and large, most of the decorative paints sold in developing countries are produced by larger regional or national companies, he says.

But one major international company also was recently implicated. In 2011 IPEN’s partners found lead levels as high as 500,000 ppm in paints produced in Cameroon by Seigneurie, an acquired subsidiary of Pittsburgh-based PPG, one of the world’s largest paint manufacturers.^40^ The partners brought the findings to PPG’s attention. PPG spokesman Mark Silvey says the company’s subsidiary reformulated its consumer coatings to meet the U.S. standard of 90 ppm as of late 2011. Silvey says the company “does not manufacture, sell, or market any architectural paint or decorative coatings that contain lead compounds anywhere in the world,” adding that PPG supports Cameroon setting a standard for lead in consumer paints.

Perry Gottesfeld, executive director of the San Francisco-based NGO Occupational Knowledge International, a GAELP member and IPEN partner, helped conduct the survey of 61 Cameroonian paints that turned up the high lead levels in Seigneurie and other brands. He says that after considerable negotiation, Seigneurie agreed to recall old lead-based paints that were still on store shelves, and that in March 2014 his Cameroonian partners confirmed the company had done so, at least for some of its old paints.

“This is the first case that we know of where a company has actually taken the stuff back, not to resell it but to actually dispose of it,” says Gottesfeld. “It’s key that companies not just reformulate their product but also take harmful product off the shelf to keep it away from consumers who are going to be harmed by the presence of lead paint in their homes. And this is particularly true where these products are not even labeled as having contained lead.”

U.S. companies sell lead compounds abroad that have potential uses in paints. In 2013 U.S. firms exported 7,400 tons of two lead oxides, red lead and orange lead, valued at around $18 million.^41^ According to Weinberg, these pigments are used in industrial paints, as well as in some anti-corrosion paints sold to consumers, but generally are not used in residential paints. Three pigments that are—lead chromate, lead sulfochromate, and lead chromate molybdate sulphate—will be restricted in European Union nations effective in 2015.^42^

Ultimately GAELP aims to minimize and prevent exposures to lead in all paints—not just the decorative paints that endanger children the most, but also industrial and automotive paints. These pose a risk to workers, as well as, potentially, to ordinary people after the paints wear and enter the environment.^43^ Australia alone restricts lead in such paints; the European Union will do so in 2015 and the Philippines in 2019.

Only a single major paint company, Amsterdam-based AkzoNobel, says it has eliminated lead from its entire product line, a change completed in late 2011, according to company spokesman Jeroen Pul. The company has called on other major paint manufacturers to follow its example, and has written to all the trade associations of which it is a member “asking to discuss the prospects for a voluntary industry agreement to phase out lead,” Pul says. He adds, “Given that effective pigments and driers that do not contain lead are now widely available, there is no need or justification to add lead compounds to paint.”

## A Weight on Health and Society

Globally, children’s blood lead levels have declined substantially, largely due to the elimination of leaded gasoline in most countries.^44^ Nevertheless, 49% of all children and 42% of adults have blood lead levels above 5 µg/dL, and lead exposures from paints, gasoline exhaust residue, mining, battery recycling, and other sources contribute to 600,000 new cases of intellectual disabilities every year, according to the WHO.^45^^,^^46^ Carolyn Vickers, who represents the WHO on GAELP, says these other major sources of lead poisoning also need urgent and concerted action, particularly in developing countries, which are home to 90% of children with elevated blood lead levels.

The societal burden of lead may include numerous social problems such as increased criminality and violence.^5^^,^^47^^,^^48^ In addition, a recent study estimated that the population-wide loss of IQ points resulting from lead exposure costs low- and middle-income countries $977 billion annually in decreased productivity, with each IQ point lost costing a child an estimated 2% of their lifetime earning potential.^49^ That economic drag amounts to 1.2% of global gross domestic product for 2011, yet the cost is nearly invisible unless you crunch the numbers, says lead author Leonardo Trasande, an associate professor at New York University.

“If a child comes back with one IQ point loss, the parent doesn’t notice. But if 100,000 kids come back with one less IQ point, the economy notices,” Trasande says, adding that for some countries, the average number of IQ points lost to lead exposure is much higher than 1. Clearly, Trasande says, the numbers justify devoting more money and effort to eradicating lead-based paints and other lead exposure sources.

Yet, as in South Africa, many countries simply lack the trained personnel, basic health infrastructure, and equipment needed to test for lead exposure, let alone screen populations comprehensively, says Vickers, who is helping to develop WHO guidelines on the prevention and medical treatment of lead exposure. She says doctors may be unaware of the issue or occupied with more glaring health needs.

In Nepal, for example, researchers reportedly conducted the first study of children’s blood lead levels in 2013 and have yet to release the results. Nepalese pediatricians generally diagnose lead poisoning only in rare instances, when it is severe enough to cause obvious neurological problems or bluish stains on the gums, says Jyoti Dhakhwa, head of the Nepal Paediatric Society. The society is currently focused on more immediate problems such as immunization campaigns and child abuse. “Many of us don’t consider lead poisoning as a serious problem,” Dhakhwa says.

However, Trasande’s study estimated that lead exposure zaps more than US$1.5 billion from Nepal’s economy each year, equivalent to 4% of gross domestic product.^49^ That stark figure, along with continued findings of high lead levels in paints on market shelves, helped convince the Nepalese government to start developing regulations for lead in paints, says Ram Charitra Sah. “The country cannot go forever with that kind of huge economic loss, which is never going to be compensated by any means,” he says.

## Cleaning Up

In the United States, the last drop of lead-based residential paint was manufactured 36 years ago.^19^ Yet for all its wealth compared with the rest of the world, the country is still struggling to overcome its historic use of the paints. As of 2006, an estimated 22% of U.S. homes—23.2 million of them—still contained lead-based paint hazards.^50^ And an analysis of data from the 2007–2010 National Health and Nutrition Examination Survey indicated 535,000 young children could have unsafe blood lead levels at or above 5 µg/dL.^51^ In an earlier paper, Trasande estimated that the United States forfeits $50.9 billion in economic activity each year because of IQ points lost to lead exposure.^52^

Even so, the country has wavered in its resolve to address the problem. Congress cut the budget for the Healthy Homes and Lead Poisoning Prevention program at the Centers for Disease Control and Prevention (CDC) from $29 million in fiscal year 2011 to $2 million in fiscal year 2012. This significantly cut funding that states relied on for screening, intervention, and cleanup of contaminated homes.^53^ To the further dismay of many in the public health community, in October 2013 the CDC eliminated an influential 25-year-old scientific committee that advised it on lead-related matters.^54^ Some relief came in January 2014 when Congress restored part of the CDC program’s budget, to $15 million.^55^

Dominique Kpokro, program director of GAELP member and IPEN partner Jeunes Volontaires pour l’Environnement, has been working toward a ban on lead-based paints in Côte d’Ivoire. He has been watching the expensive, unfinished cleanup effort in the United States and is mindful that poor nations will find it all but impossible to do even that—all the more reason to abolish lead-based paints now, he says.

IPEN’s Weinberg says eliminating lead in decorative paints by 2020 has become a personal goal, one he came out of retirement to accomplish. Additional funding would speed up the job considerably, he says, and there is no question a global effort could succeed with even a relatively modest but consistent stream of resources. “But this is not yet assured, and this promising global effort could still stall,” he says. If so, he warns, the world community could again forget about lead-based paints for another 40 years.

On the other hand, Weinberg says, momentum is building as countries act more quickly once their neighbors have gone lead-free and suppliers are increasingly able to provide competitively priced lead-free ingredients. “In terms of cost effectiveness, bang for your buck, eliminating lead paint is about the cheapest public health intervention with the greatest public health benefit imaginable,” he says.^56^ “We’ll do it. … I am certain we will succeed.”
